# Quantitative interactome proteomics identifies a proteostasis network for GABA_A_ receptors

**DOI:** 10.1016/j.jbc.2022.102423

**Published:** 2022-08-27

**Authors:** Ya-Juan Wang, Xiao-Jing Di, Ting-Wei Mu

**Affiliations:** 1 Department of Physiology and Biophysics, Case Western Reserve University School of Medicine, Cleveland, Ohio, USA; 2 Center for Proteomics and Bioinformatics, Case Western Reserve University School of Medicine, Cleveland, Ohio, USA

**Keywords:** GABA_A_ receptor, epilepsy, SILAC, interactome, proteostasis, folding, assembly, trafficking, degradation, ABC, ammonium bicarbonate, ACN, acetonitrile, CANX, calnexin, CHX, cycloheximide, DAVID, Database for Annotation, Visualization and Integrated Discovery, DMEM, Dulbecco’s modified Eagle's medium, ER, endoplasmic reticulum, ERAD, ER-associated degradation, EV, empty vector, FA, formic acid, FBS, fetal bovine serum, FDR, false discovery rate, GABA_A_, gamma-aminobutyric acid type A, GO, Gene Ontology, HEK293T, human embryonic kidney 293T cell line, IP–MS/MS, immunoprecipitation–tandem mass spectrometry, MS, mass spectrometry, QC, quality control, *RCN2*, reticulocalbin 2, SILAC, stable isotope labeling by amino acids in cell culture, SRP, signal recognition particle, TM, transmembrane, TRIM21, tripartite motif containing-21, UGGT1, UDP-glucose glycoprotein glucosyltransferase 1

## Abstract

Gamma-aminobutyric acid type A (GABA_A_) receptors are the primary inhibitory neurotransmitter-gated ion channels in the mammalian central nervous system. Maintenance of GABA_A_ receptor protein homeostasis (proteostasis) in cells utilizing its interacting proteins is essential for the function of GABA_A_ receptors. However, how the proteostasis network orchestrates GABA_A_ receptor biogenesis in the endoplasmic reticulum is not well understood. Here, we employed a proteomics-based approach to systematically identify the interactomes of GABA_A_ receptors. We carried out a quantitative immunoprecipitation-tandem mass spectrometry analysis utilizing stable isotope labeling by amino acids in cell culture. Furthermore, we performed comparative proteomics by using both WT α1 subunit and a misfolding-prone α1 subunit carrying the A322D variant as the bait proteins. We identified 125 interactors for WT α1-containing receptors, 105 proteins for α1(A322D)-containing receptors, and 54 overlapping proteins within these two interactomes. Our bioinformatics analysis identified potential GABA_A_ receptor proteostasis network components, including chaperones, folding enzymes, trafficking factors, and degradation factors, and we assembled a model of their potential involvement in the cellular folding, degradation, and trafficking pathways for GABA_A_ receptors. In addition, we verified endogenous interactions between α1 subunits and selected interactors by using coimmunoprecipitation in mouse brain homogenates. Moreover, we showed that TRIM21 (tripartite motif containing-21), an E3 ubiquitin ligase, positively regulated the degradation of misfolding-prone α1(A322D) subunits selectively. This study paves the way for understanding the molecular mechanisms as well as fine-tuning of GABA_A_ receptor proteostasis to ameliorate related neurological diseases such as epilepsy.

Normal organismal physiology depends on the maintenance of protein homeostasis (proteostasis) in each cellular compartment (1, 2, 3, 4), which dictates a delicate balance between protein synthesis, folding, assembly, trafficking, and degradation while minimizing misfolding and aggregation (5, 6, 7). For one specific client protein, its interaction with a network of proteins, especially its proteostasis network components, in the crowded cellular environment is critical to maintain its proteostasis. However, how the proteostasis network orchestrates the biogenesis of multisubunit multispan ion channel proteins is poorly understood. The current limited knowledge about such protein quality control (QC) machinery is gained from the study of various classes of membrane proteins, including cystic fibrosis transmembrane (TM) conductance regulator (8), T-cell receptors (9), sodium channels (10), potassium channels (11), and nicotinic acetylcholine receptors (12). We have been using gamma-aminobutyric acid type A (GABA_A_) receptors as an important membrane protein substrate to clarify its biogenesis pathway (13), which is currently understudied.

GABA_A_ receptors are the primary inhibitory neurotransmitter-gated ion channels in mammalian central nervous systems (14) and provide most of the inhibitory tone to balance the tendency of excitatory neural circuits to induce hyperexcitability, thus maintaining the excitatory–inhibitory balance (15). There are 19 known GABA_A_ receptor subunits in mammals, including α1–6, β1–3, γ1–3, θ, ε, π, δ, and ρ1–3. Loss of function of GABA_A_ receptors is a prominent cause of genetic epilepsies, and recent advances in genetics have identified a growing number of epilepsy-associated variants in over ten genes that encode the subunits of GABA_A_ receptors, including over 150 variants in those encoding major synaptic subunits (α1, β2, and γ2 subunits) (Fig. 1*A*, *top right figure*) (16, 17, 18, 19, 20). GABA_A_ receptors belong to the Cys-loop superfamily of ligand-gated ion channels, sharing common structural characteristics with other Cys-loop receptor members (21). A functional GABA_A_ receptor is composed of five subunits. Each subunit has a large extracellular (or the endoplasmic reticulum [ER] luminal) N terminus, four TM helices (TM1–TM4, with TM2 domain lining the interior of the pore), and a short extracellular (or the ER luminal) C terminus (Fig. 1*A*) (22, 23). GABA binding to GABA_A_ receptors in the extracellular domain induces conformational changes, opens the ion pore to conduct chloride, hyperpolarizes the plasma membrane, and inhibits neuronal firing in mature neurons.

**Figure 1:**
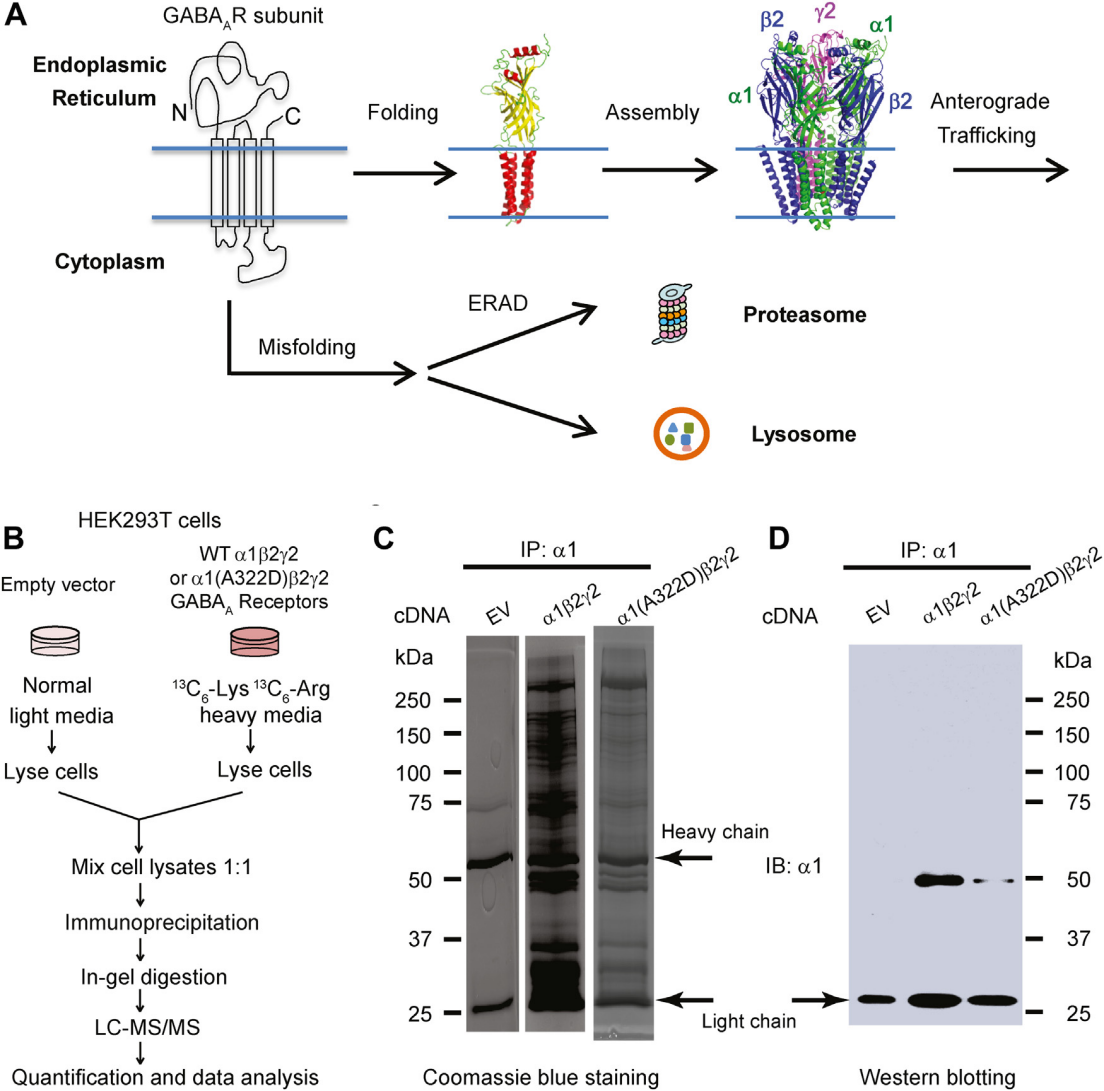
**Quantitative immunoprecipitation–tandem mass spectrometry (IP–MS/MS) analysis to identify GABA**_**A**_**receptor interactomes.***A*, GABA_A_ receptor biogenesis pathways. Individual subunits of GABA_A_ receptors fold in the endoplasmic reticulum (ER). Properly folded subunits assemble into a heteropentamer in the ER for subsequent trafficking to the plasma membrane. Unassembled and misfolded subunits are subjected to the ER-associated degradation (ERAD) pathway by the proteasome or the lysosome-related degradation. *B*, outline of a comparative SILAC-based quantitative proteomics approach to identify GABA_A_ receptor interactomes in HEK293T cells. The α1(A322D) variant leads to its excessive misfolding and ERAD. *C* and *D*, HEK293T cells expressing empty vector (EV), WT, or α1(A322D)β2γ2 GABA_A_ receptors were subjected to coimmunoprecipitation using an anti-GABA_A_ receptor α1 subunit antibody. The immunoisolated complexes were separated by SDS-PAGE and visualized by Coomassie blue staining (*C*) or Western blot analysis (*D*). Both heavy chain and light chain bands were detected in Coomassie blue gels, whereas only light chain bands were detected in Western blot because of the usage of a light chain–specific secondary antibody for the detection. GABA_A_, gamma-aminobutyric acid type A; HEK293T, human embryonic kidney 293T cell line; IB, immunoblotting; IP, immunoprecipitation; SILAC, stable isotope labeling by amino acids in cell culture.

To function properly, GABA_A_ receptors need to fold into their native structures and assemble correctly to form a pentamer on the ER membrane and traffic efficiently through the Golgi en route to the plasma membrane (Fig. 1*A*). Misfolded GABA_A_ receptors are recognized by the cellular protein QC machinery. ER-associated degradation (ERAD) is one major cellular pathway to target misfolded GABA_A_ receptors to the cytosolic proteasome for degradation (7, 24, 25, 26, 27). Another potential degradation pathway is to target the aggregation-prone GABA_A_ receptors to the lysosome through autophagy, ER-phagy, or ER-to-lysosome-associated degradation (28, 29, 30, 31). Maintenance of a delicate balance between GABA_A_ receptor folding, trafficking, and degradation utilizing its interacting proteins is critical for its function. However, the GABA_A_ receptor interactome, especially the proteostasis network that orchestrates GABA_A_ receptor biogenesis in the ER, has not been studied systematically in the literature despite recent advances about the trafficking of GABA_A_ receptors beyond the ER (32, 33, 34, 35, 36, 37). Here, we used quantitative immunoprecipitation–tandem mass spectrometry (IP–MS/MS) analysis utilizing stable isotope labeling by amino acids in cell culture (SILAC) in human embryonic kidney 293T (HEK293T) cells to identify the interactomes for both WT and a misfolding-prone GABA_A_ receptor. Endogenous interactions between selected interactors and GABA_A_ receptors were verified in mouse brain homogenates. Furthermore, bioinformatics analysis enabled us to assemble a proteostasis network model for the cellular folding, assembly, degradation, and trafficking pathways of GABA_A_ receptors.

## Results

### Identification of interactomes for WT and misfolding-prone GABA_A_ receptors using comparative SILAC-based proteomics

We employed a proteomics-based approach to identify the interactomes of GABA_A_ receptors in HEK293T cells by carrying out a quantitative IP–MS/MS analysis utilizing SILAC (Fig. 1*B*) (38). Since HEK293T cells do not own endogenous GABA_A_ receptors, precise control of the subtypes and variants of GABA_A_ receptors can be achieved by exogenously expressing their subunits (39). Furthermore, we performed comparative proteomics by using both WT α1 subunit and a well-characterized misfolding-prone α1 subunit carrying the A322D variant as the bait proteins to determine the potential difference between them (40). The A322D variant introduces an extra negative charge in the third TM (TM3) helix of the α1 subunit, causing its substantial misfolding and excessive degradation by ERAD (40, 41). HEK293T cells stably expressing either WT α1β2γ2 or α1(A322D)β2γ2 GABA_A_ receptors were labeled with heavy media, whereas HEK293T cells that were transfected with empty vector (EV) plasmids were cultured in normal light media. The same amount of light and heavy cell lysates was mixed. The α1 or α1(A322D) complexes were immunoprecipitated using a monoclonal antibody against the N-terminal region of the α1 subunit before being subjected to SDS-PAGE and in-gel digestion and tandem MS analysis. Coomassie blue–stained gels showed numerous clearly visible bands in samples expressing GABA_A_ receptors (Fig. 1*C*, lanes 2 and 3), which represented potential interacting proteins for α1 subunit–containing GABA_A_ receptors, indicating efficient coimmunoprecipitations. In addition, Western blot analysis detected α1 subunits effectively at ∼50 kDa postimmunoprecipitation in samples expressing GABA_A_ receptors (Fig. 1*D*, lanes 2 and 3), whereas no α1 band was observed in the EV control sample (Fig. 1*D*, lane 1), supporting the efficient isolation of the α1 subunit–containing GABA_A_ receptor complexes.

**Figure 2:**
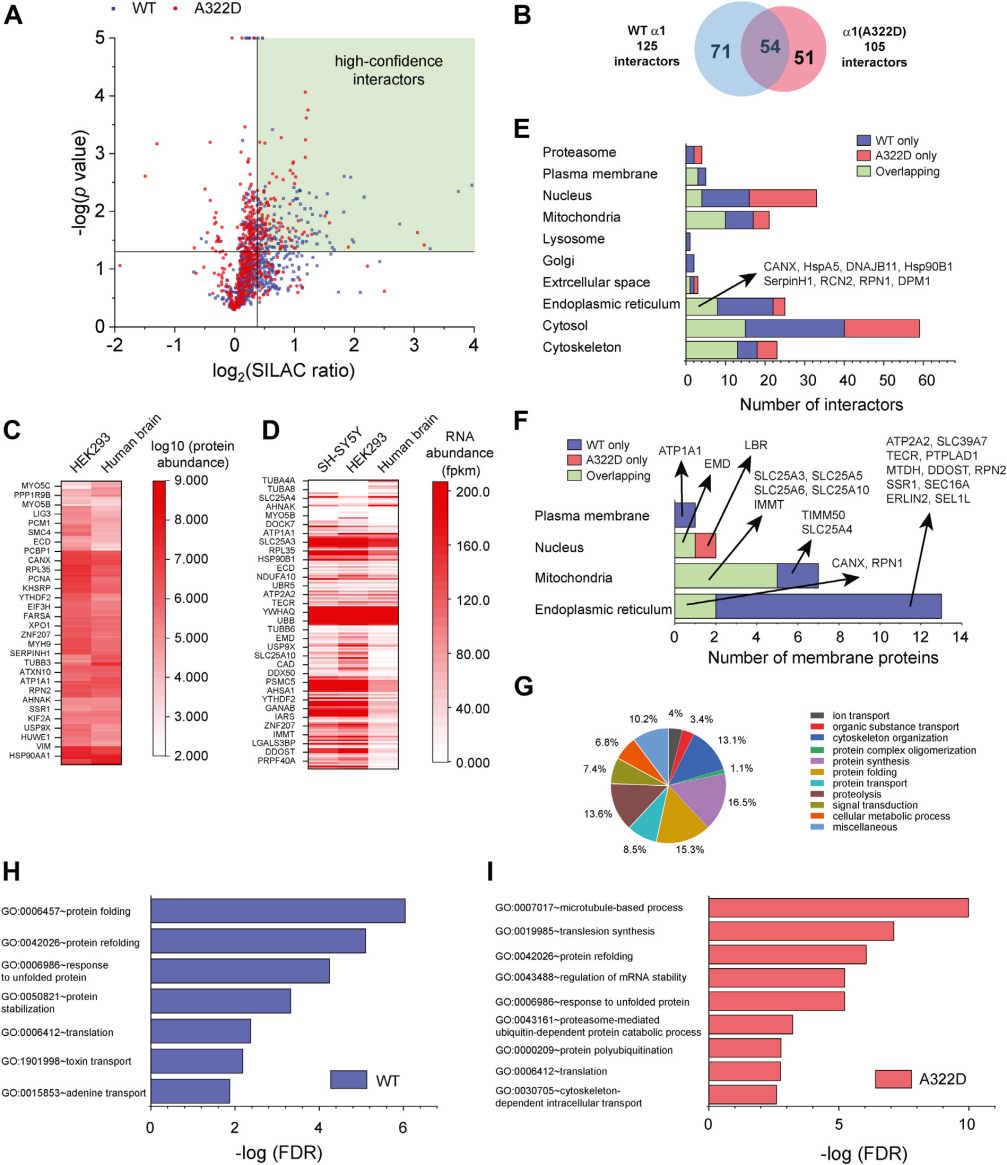
**Bioinformatics analysis of GABA**_**A**_**receptor interactomes.***A*, a 2D plot showing the relationship between *p* value and SILAC ratio for proteins that were identified from tandem MS analysis. Proteins that are in the WT samples are colored in *blue*; those in α1(A322D) variant–containing GABA_A_ receptor samples, in *red*. The *vertical black line* represents a SILAC ratio of 1.30, whereas the *horizontal black line* represents a *p* value of 0.05. *B*, a Venn diagram showing the protein numbers and overlap of the GABA_A_ receptor interactomes. *C*, a heat map showing the protein abundance for GABA_A_ receptor interactors in HEK293 cells and human brain tissues. *D*, a heat map showing the RNA abundance for GABA_A_ receptor interactors in HEK293 cells, SH-SY5Y cells, and human brain tissues. *E*, analysis of cellular components for GABA_A_ receptor interactomes. *F*, analysis of cellular components for GABA_A_ receptor interactors that are integral membrane proteins. *G*, a pie chart showing the biological processes of the pooled 176 GABA_A_ receptor interactors. Enriched biological processes for WT α1-containing GABA_A_ receptor interactome (*H*) and α1(A322D)-containing GABA_A_ receptor interactome (*I*) according to DAVID analysis. DAVID, Database for Annotation, Visualization and Integrated Discovery; FDR, false discovery rate. Fpkm, fragment per kilobase of exon per million mapped fragment; GABA_A_, gamma-aminobutyric acid type A; HEK293, human embryonic kidney 293 cell line; MS, mass spectrometry; SILAC, stable isotope labeling by amino acids in cell culture.

The α1 subunit–containing GABA_A_ receptor interactomes were identified using the SILAC ratio with arbitrary yet strict criteria to remove potential false positives. To be included as an interactor, it must (1) have a SILAC ratio of WT α1/EV or α1(A322D)/EV to be at least 1.30; (2) have a *p* < 0.05; and (3) have a Benjamini and Hochberg correction (42) of false discovery rate (FDR) of no more than 0.10. The *top right green* area in Figure 2, *A* contains high-confidence interactors for GABA_A_ receptors. As a result of the stringent criteria, the WT α1-containing GABA_A_ receptor interactome contains 125 proteins, the α1(A322D)-containing GABA_A_ receptor interactome contains 105 proteins, and 54 proteins overlap within two interactomes (Fig. 2*B*). These 176 interactors for GABA_A_ receptors are used for the following bioinformatics analysis (see Table S1 for protein list).

### Comparison with previous GABA_A_ receptor proteomic analyses

Since GABA_A_ receptors inhibit neuronal firing in the mammalian central nervous system, we first compared the expression abundance of their interactors between HEK293 cells and the nervous system. ProteomicsDB (https://www.proteomicsdb.org/) provides an MS-based navigation of the human proteome from tissues, cell lines, and body fluids, enabling the comprehensive mapping of the protein abundance of GABA_A_ receptor interactors (43). Hierarchical clustering analysis showed that these interactors have comparable protein levels between HEK293 cells and human brain tissues (Fig. 2*C* and Table S2). For example, many molecular chaperones, such as Hsp90s (*Hsp90AA1*, *Hsp90AB1*, and *Hsp90B1*), Hsp70s (*HspA5* and *HspA8*), Hsp40s (*DNAJA1*, *DNAJA2*, and *DNAJB11*), calnexin (*CANX*), and Hsp47 (*SerpinH1*), are abundantly expressed in both systems. In addition, tissue-based map of the human proteome based on quantitative transcriptomics (https://www.proteinatlas.org/) enabled us to compare RNA levels of GABA_A_ receptor interactors between HEK293 cells, a human neuronal SH-SY5Y cell line, and human brain tissues (44, 45). Hierarchical clustering analysis showed that many interactors, including molecular chaperones and ubiquitin-dependent degradation factors, such as *UBA1*, *UBR5*, *UBE3C*, *SEL1L*, and *VCP*, have similar RNA expression patterns between these systems (Fig. 2*D* and Table S2). These results are consistent with the report that most proteins are well conserved among human tissues (45).

Previously, proteomic analyses were carried out to identify the interacting proteins for GABA_A_ receptor subunits using knockin mice carrying Venus (a GFP variant)-tagged α1 subunit (37), pHluorin (a pH-sensitive GFP variant)-Myc-tagged α2 subunit (36), or His_6_-FLAG-YFP-tagged γ2 subunit (32). GFP-affinity purification from the cerebral cortex using Venus-tagged α1 subunits as bait led to the identification of 18 proteins in the inhibitory synaptic complexes, including 11 GABA_A_ receptor subunits, five scaffolding and adhesion proteins (gephrin [*GPHN*], neuroligin 2 [*NLGN2*], neuroligin 3 [*NLGN3*], collybistin [*ARHGEF9*], and neurexin 1 [*NRXN1*]), and two proteins with less known functions (neurobeachin [*NBEA*] and LHFPL4) (37). However, because of the use of size-exclusion chromatography during the protein complex purification process, proteins that are potentially involved in the cellular folding and trafficking of GABA_A_ receptors were not well identified.

GFP-trap purification from hippocampus and cortex using pHluorin-Myc-tagged α2 subunits as bait resulted in the identification of 174 proteins, including 14 GABA_A_ receptor subunits, known GABA_A_ receptor interactors such as gephrin, neuroligins, and collybistin, and 149 novel binding partners (36). The novel interactors were further categorized into five groups, including G protein–coupled receptors, ion channels, and transporters (26 interactors), factors that regulate protein trafficking, stability, and cytoskeletal anchoring (38 interactors), factors that regulate phosphorylation and GTP exchange (26 interactors), miscellaneous enzymes (27 interactors), and miscellaneous proteins (32 interactors) (36). Comparison between the α2 subunit–containing GABA_A_ receptor interactome and our α1 subunit–containing GABA_A_ receptor interactome showed overlapping interactors, including factors that regulate protein folding and degradation (*DNAJA1*, *RPN2*, *SQSTM1*, *USP9X*, and *DDB1*), transporters (*SLC25A3*, *SLC25A4*, and *SLC25A5*), and miscellaneous proteins (*PHB2* and *IMMT*).

Furthermore, tandem affinity purification from whole brain homogenates using His_6_-FLAG-YFP-tagged γ2 subunits as bait gave rise to the identification of 11 known associated proteins (GABA_A_ receptor subunits, gephrin and neuroligin 2) and 39 novel binding partners (32). The novel interactors were further classified into five groups, including ion channels (two interactors), factors that regulate protein folding and trafficking (six interactors), mitochondria proteins (six interactors), miscellaneous enzymes (seven interactors), and miscellaneous proteins (18 interactors). Comparison between the γ2 subunit–containing GABA_A_ receptor interactome and our α1 subunit–containing GABA_A_ receptor interactome showed overlapping interactors, including a factor that regulate protein degradation (*PSMC2*), an enzyme (*PTPLAD1*), and a miscellaneous protein (*EMD*).

Among the three GABA_A_ receptor proteomic analyses using knockin mice, six synaptic proteins were recognized from at least two studies, including gephrin, neuroligin 2, neuroligin 3, collybistin, neurobeachin, and LHFPL4. However, such synaptic proteins were not identified in our α1 subunit–containing GABA_A_ receptor interactome possibly because of their lack of expression in HEK293T cells. The only other overlapping interactor from at least two knockin mice proteomic studies is CACNA1E, the α1E subunit of R-type voltage-dependent calcium channels, which could indicate that different subunits utilize differentiating cellular interaction networks. Moreover, the limited interactome overlapping from these proteomic analyses could arise from using different purification procedures to isolate the GABA_A_ receptor–containing complex, such as using different detergents. Nonetheless, in addition to certain GABA_A_ receptor subunits, our α1 subunit–containing GABA_A_ receptor interactome showed 13 overlapping interactors with one other proteomic study, providing promising candidates for future investigation.

### Gene Ontology analysis of GABA_A_ receptor interactomes

To annotate cellular component for GABA_A_ receptor interactors, we used Database for Annotation, Visualization and Integrated Discovery (DAVID) to carry out Gene Ontology (GO) analysis (46, 47). Since many proteins reside in more than one cellular location, we only choose one primary subcellular location for them manually. To aid such an assignment, we also integrate subcellular location information from UniProt Database (https://www.uniprot.org/) and GeneCards: The Human Gene Database (https://www.genecards.org/). GABA_A_ receptor interactors are distributed in various cellular locations, including the nucleus (33 interactors), ER (25 interactors), Golgi (2 interactors), proteasome (4 interactors), lysosome (1 interactor), mitochondria (21 interactors), cytoskeleton (23 interactors), cytosol (59 interactors), plasma membrane (5 interactors), and extracellular space (3 interactors) since biogenesis and function of GABA_A_ receptors require their interactions with a network of proteins throughout the cell (Fig. 2*E* and Table S3). Because of the essential role of the ER in protein QC, 25 interactors (14 for WT α1-containing GABA_A_ receptor only, three for α1(A322D) variant–containing GABA_A_ receptor only, and eight for both) are located to this organelle. Eight overlapping interactors in the ER include molecular chaperones (*CANX*, *HspA5*, *DNAJB11*, *Hsp90B1*, and *SerpinH1*), factors involved in *N*-linked glycosylation (*RPN1* and *DPM1*), and a Ca^2+^-binding protein (reticulocalbin 2 [*RCN2*]). *RCN2* (aka *ERC55*) encodes RCN2 in the ER lumen; it is abundantly expressed in the brain and contains EF-hand Ca^2+^-binding motifs (48). Function of RCN2 is largely unknown, although gene expression of *RCN2* is upregulated in patients with idiopathic absence epilepsies (49). Given the critical role of GABA_A_ receptors in the pathophysiology of epilepsy, it will be of great interest to determine how RCN2 regulates GABA_A_ receptor biogenesis and contributes to epilepsy phenotypes in the future.

Furthermore, since GABA_A_ receptors are TM proteins, we extracted their interactors that are integral membrane proteins, including 13 in the ER, seven in the mitochondria (*SLC25A3*, *SLC25A4*, *SLC25A5*, *SLC25A6*, *SLC25A10*, *IMMT*, and *TIMM50*), two in the nucleus (*EMD* and *LBR*), and one in the plasma membrane (*ATP1A1*) (Fig. 2*F* and Table S3). *ATP1A1* encodes α1 subunit of sodium/potassium-transporting ATPase in the plasma membrane. Variants in *ATP1A1* cause hypomagnesemia, seizures, and mental retardation 2, an autosomal dominant disease characterized by generalized seizures in infancy and significant intellectual disability (50). The 13 membrane interactors in the ER include molecular chaperones and folding enzymes (*CANX*, *DDOST*, *RPN1*, and *RPN2*), trafficking factors (*SSR1* and *SEC16A*), ERAD factors (*ERLIN2* and *SEL1L*), transporters (*ATP2A2* and *SLC39A7*), enzymes involved in the lipid metabolism (*TECR* and *PTPLAD1*), and other (*MTDH*). Since each subunit of GABA_A_ receptors has four TM helices, these ER membrane interactors have the potential to form intramembrane interactions with GABA_A_ receptors to regulate their biogenesis in the ER, which needs to be explored in the future. Since the extensive dynamic ER network can form membrane contact sites with a variety of organelles, including the mitochondria (51), seven mitochondria membrane interactors were identified for GABA_A_ receptors. Interestingly, SLC25A3, SLC25A4, SLC25A5, and IMMT were also identified from a previous proteomic study using the pHluorin-Myc-tagged α2 subunit as a bait protein in the knockin mice (36). Although these seven mitochondria membrane interactors are annotated as inner membrane proteins, they could either utilize other adapter proteins or own cytosolic components to be involved in the ER–mitochondria interactions.

Moreover, we used DAVID, UniProt, and GeneCards to annotate biological process for GABA_A_ receptor interactors. The 176 interactors were annotated to the following functional categories: ion transport (4%), organic substance transport (3.4%), cytoskeleton organization (13.1%), protein complex oligomerization (1.1%), protein synthesis (16.5%), protein folding (15.3%), protein transport (8.5%), proteolysis (13.6%), signal transduction (7.4%), cellular metabolic process (6.8%), and miscellaneous (10.2%) (Fig. 2*G* and Table S4). Furthermore, we used DAVID to determine enriched biological process for GABA_A_ receptor interactors. Both WT α1-containing GABA_A_ receptor interactors and α1(A322D)-containing GABA_A_ receptor interactors form five functional clusters (Table S5). Top enriched biological processes from those clusters are plotted in Figure 2, *H* and *I* according to FDR. Protein refolding (GO: 0042026), response to unfolded protein (GO: 0006986), and translation (GO: 0006412) are common enriched biological processes for both WT and α1(A322D) interactomes. Since the A322D variant causes extensive protein misfolding and ERAD of the α1 subunit, the α1(A322D)-containing GABA_A_ receptor interactome enriches proteasome-mediated ubiquitin-dependent protein catabolic process (GO: 0043161) and protein polyubiquitination (GO: 0000209).

### Mapping of the proteostasis network for GABA_A_ receptors

**Figure 3:**
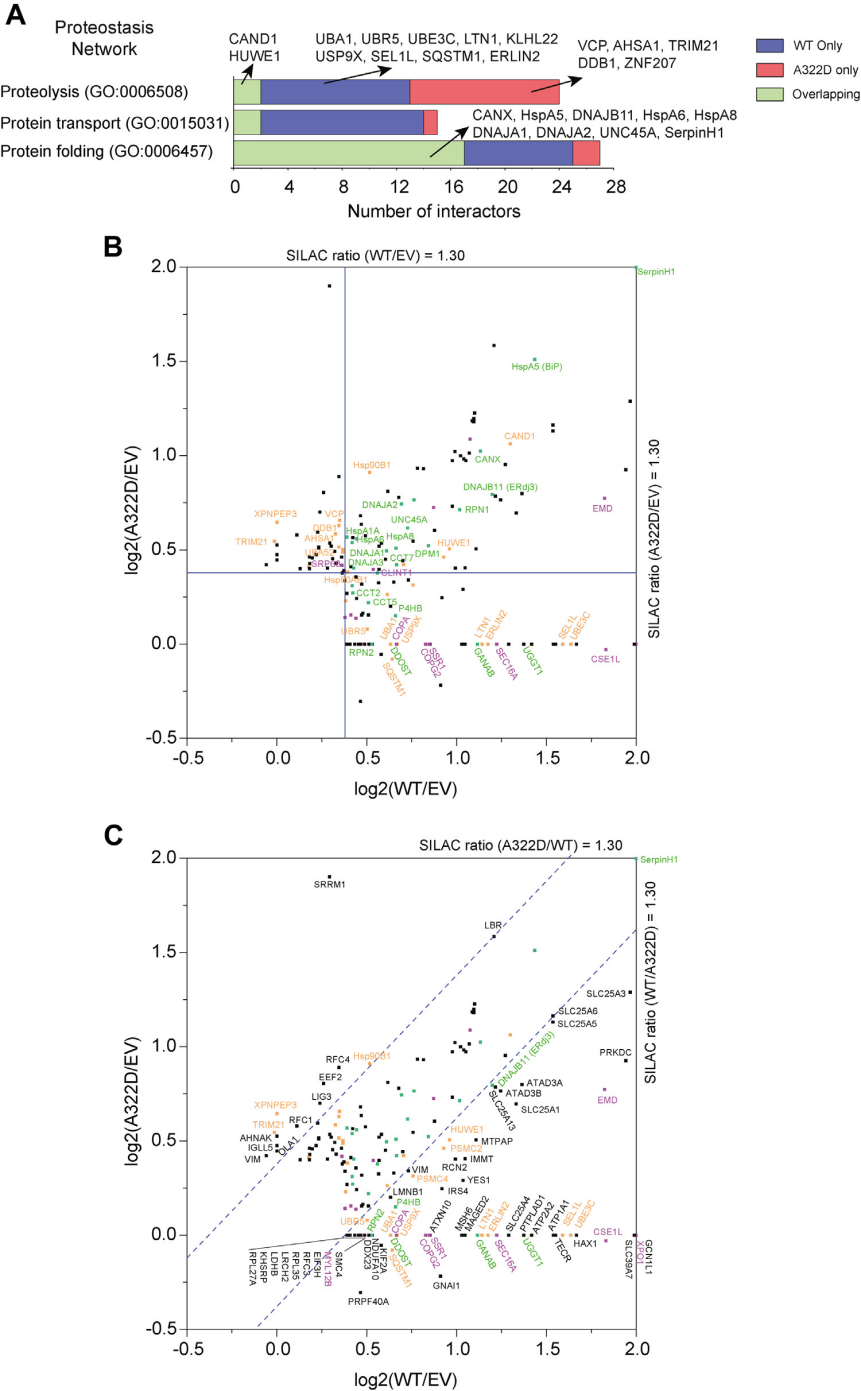
**Proteome profiling of proteostasis network for WT and misfolding-prone α1(A322D)-containing GABA**_**A**_**receptors.***A*, Gene Ontology analysis of the proteostasis network for GABA_A_ receptor interactomes. Number of interactors is plotted against proteolysis, protein transport, and protein folding. *B* and *C*, a 2D plot showing SILAC ratios of the identified interactors. The *x*- and *y*-axes show log_2_(SILAC ratio of WT α1/EV) and log_2_(SILAC ratio of α1(A322D)/EV). For display purpose, if the SILAC ratio is greater than 4.0, it is displayed as 4.0; if a protein is only detected in one sample, the SILAC ratio of this protein in the other sample is artificially set to 1.0. *Blue solid lines* represent a cutoff SILAC ratio of 1.30 to be considered as interactors for WT α1-containing GABA_A_ receptors or the α1(A322D) variant–containing GABA_A_ receptor (*B*)*.Blue dashed lines* represent a cutoff SILAC ratio of 1.30 to be considered as interactors that preferentially bind WT or α1(A322D) variant–containing GABA_A_ receptor (*C*)*.* Based on literature knowledge, interactors that are expected to play a role in the folding and assembly of GABA_A_ receptors are colored in *green*; those in their degradation, in *orange*; those in their transport, in *purple*; and those with unknown roles in proteostasis maintenance, in *black*. EV, empty vector; GABA_A_, gamma-aminobutyric acid type A; SILAC, stable isotope labeling by amino acids in cell culture.

We focused on identifying the proteostasis network for GABA_A_ receptors, which regulates their folding, assembly, trafficking, and degradation. Based on GO analysis and literature knowledge, we assigned the interactors to proteostasis network categories, including protein folding (GO: 0006457), proteolysis (GO: 0006508), and protein transport (GO: 0015031) (Table S6). The proteostasis network components account for 37.4% of the total interactors, indicating their critical role in regulating GABA_A_ receptor function. The number of interactors that belong to the proteostasis network was plotted for WT α1-containing GABA_A_ receptor interactome, α1(A322D)-containing GABA_A_ receptor interactome, and overlapping interactome (Fig. 3*A*). In addition, we visualized the proteostasis network for WT and α1(A322D) variant–containing receptors in a 2D plot showing their SILAC ratios (Fig. 3*B* and Table S1).

Judging from the literature knowledge, interactors that are expected to regulate the folding and assembly of α1 subunit–containing GABA_A_ receptors are indicated in *green*, those for protein transport in *purple*, and those for proteolysis in *orange* (Fig. 3*B*). Major chaperone networks and folding enzymes were identified from GABA_A_ receptor interactomes. These include Hsp70s and their cochaperone Hsp40s in the ER (HspA5 and DNAJB11) and in the cytosol (HspA6, HspA8, HspA1A, DNAJA1, DNAJA2, and DNAJA3), Hsp90s and their cochaperones in the ER (Hsp90B1) and in the cytosol (Hsp90AB1, AHSA1, and UNC45A), Hsp60 subunits (CCT2, CCT5, and CCT7), and a protein disulfide isomerase (P4HB). Moreover, since GABA_A_ receptors have several *N*-linked glycosylation sites, proteins that are involved in the *N*-linked glycoprotein maturation in the ER were identified, including glycosylation enzymes (DDOST, RPN1, RPN2, DPM1, GANAB, and UDP-glucose glycoprotein glucosyltransferase 1 [UGGT1]) and a lectin chaperone (CANX). Interactors that are involved in protein transport include translocon-related proteins (SSR1 and signal recognition particle 68 [SRP68]), a COPII subunit (SEC16A), COPI subunits (COPA and COPG2), and an endocytosis-related protein (CLINT1). In addition, we identified numerous proteolysis-related interactors. These include ERAD factors, including a number of ubiquitin E3 ligase complexes (HUWE1, TRIM21 [tripartite motif containing-21], UBR5, LTN1, UBE3C, CAND1, KLHL22, and DDB1), an ubiquitin E1-activating enzyme (UBA1), a deubiquitination enzyme (USP9X), retrotranslocation proteins (SEL1L and VCP), and other factors (ERLIN2).

### Comparison between interactomes of WT and misfolding-prone α1(A322D) variant–containing GABA_A_ receptors

Our proteomic analysis identified 125 interactors for WT GABA_A_ receptors and 105 interactors for misfolding-prone α1(A322D)-containing receptors, and 54 proteins overlap within these two interactomes (Fig. 2*B* and Table S1). On one hand, WT and the variant share 54 interactors (Fig. 3*B*, *top right area*) to regulate the folding, assembly, degradation, and trafficking of GABA_A_ receptors during their biogenesis in cells. Such common interactors include major chaperones and their cochaperones, such as HspA5 and DNAJB11, and CANX. On the other hand, WT GABA_A_ receptors have 71 unique interactors (Fig. 3*B*, *bottom right area*), whereas the α1(A322D)-containing receptors have 51 unique interactors (Fig. 3*B*, *top left area*), indicating that WT and the variant also utilize distinct proteostasis networks for their biogenesis. Therefore, it is feasible to target the distinct interactors to adapt the proteostasis network to achieve selectivity for the misfolding-prone variant. For example, several ERAD factors, such as VCP and TRIM21, were recognized in the *top left area* in the 2D plot (Fig. 3*B*) since the α1(A322D) protein undergoes accelerated ERAD (also see later for the selective effect of TRIM21 on the α1(A322D) degradation).

Furthermore, we defined (1) if an interactor has a SILAC ratio of α1(A322D)/WT α1 of at least 1.30, this interactor preferentially binds α1(A322D) variant–containing receptors and (2) if an interactor has a SILAC ratio of WT α1/α1(A322D) of at least 1.30, this interactor preferentially binds WT α1-containing receptors. Accordingly, 13 interactors were identified to preferentially bind α1(A322D)-containing receptors over WT (Fig. 3*C*, *top left section above the dashed line*), including three degradation factors (TRIM21, Hsp90B1, and XPNPEP3) and ten proteins with less known function in proteostasis maintenance. It is worth noting that the most enriched α1(A322D) variant–containing receptor interactor is SRRM1, encoding serine/arginine repetitive matrix protein 1. Moreover, 64 interactors were identified to preferentially bind WT α1 over α1(A322D)-containing receptors (Fig. 3*C*, *bottom right section below the dashed line*), including seven folding factors, 11 degradation factors, eight trafficking factors, and 38 proteins with less defined function in proteostasis maintenance. Top enriched WT α1-containing receptor interactors include CSE1L, GNAI1, EMD, PRKDC, GCN1L1, XPO1, SLC39A7, and HAX1, which merit future investigation.

### Verification of the role of selected GABA_A_ receptor proteostasis network components

**Figure 4:**
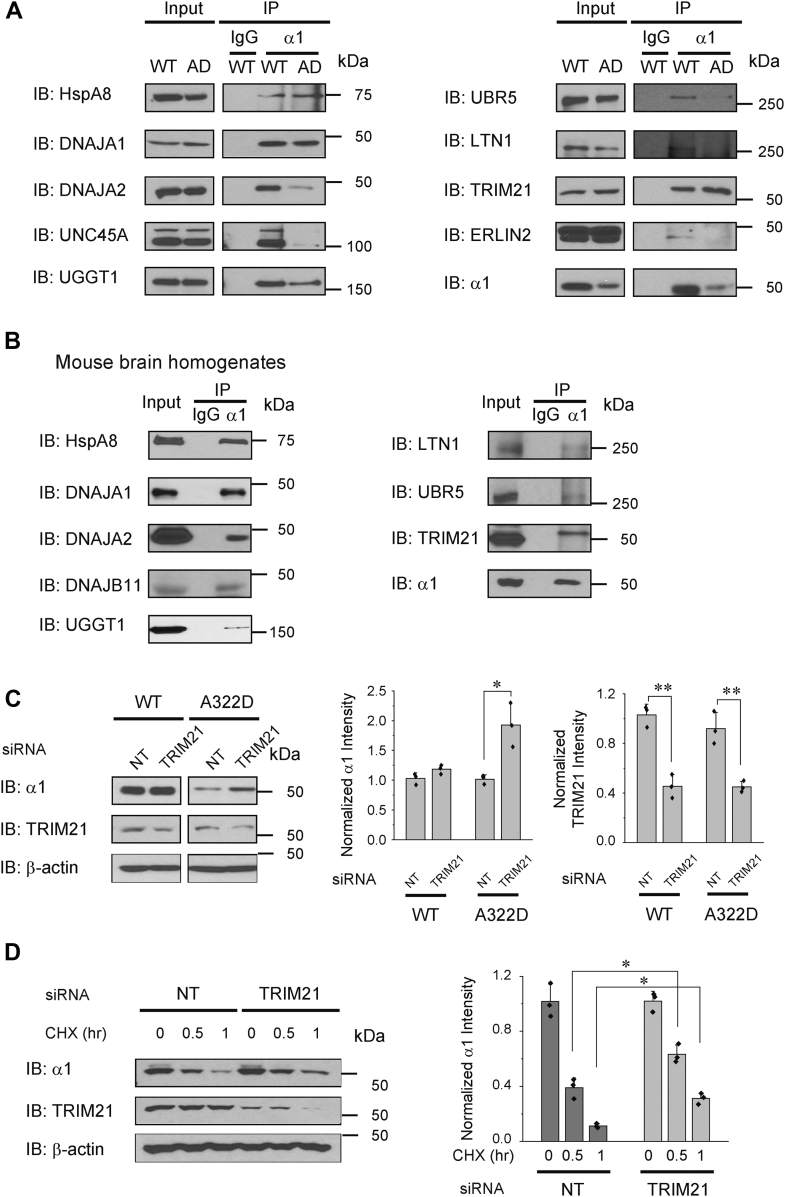
**Verification of the role of selected GABA**_**A**_**receptor interactors.** HEK293T cells stably expressing WT α1β2γ2 or α1(A322D)β2γ2 GABA_A_ receptors (*A*) or mouse brain homogenates (*B*) were immunoprecipitated using an antibody against α1 subunits and then subjected to SDS-PAGE and Western blot analysis. Three replicates were carried out. Immunoglobulin G serves as a negative control during the coimmunoprecipitation. *C*, HEK293T cells stably expressing WT α1β2γ2 or α1(A322D)β2γ2 GABA_A_ receptors were transfected with nontargeting (NT) siRNA or siRNA against TRIM21. Forty-eight hours post-transfection, cells were lysed and subjected to Western blot analysis. β-actin serves as a loading control. Quantification of the normalized protein band intensity was shown on the *right* (n = 3). Student’s *t* test was used to evaluate the statistical significance. *D*, HEK293T cells expressing α1(A322D)β2γ2 receptors were transfected as in (*C*). Cycloheximide (CHX), a potent protein synthesis inhibitor, was added to the cell culture medium for the indicated time before cell lysis and Western blot analysis. Quantification of the normalized remaining α1 band intensity was shown on the *right* (n = 3). Two-way ANOVA followed by post hoc Tukey's test was used to evaluate the statistical significance. Data are presented as mean ± SD. ∗*p* < 0.05; ∗∗*p* < 0.01. AD, α1(A322D) variant; GABA_A_, gamma-aminobutyric acid type A; HEK293T, human embryonic kidney 293T cell line; IB, immunoblotting; IP, immunoprecipitation; TRIM21, tripartite motif containing-21.

The interactions between selected GABA_A_ receptor proteostasis network components and WT α1 and α1(A322D)-containing receptors were verified using coimmunoprecipitation and Western blot analysis. We focus on molecular chaperones and ERAD factors because of their critical role in the protein QC of GABA_A_ receptors in the ER. Previously, we showed that BiP (HspA5), an Hsp70 family chaperone in the ER lumen, interacts with WT α1 and α1(A322D)-containing GABA_A_ receptors and promotes their maturation in the ER (41, 52, 53). Here, we verified that HspA8, a Hsp70 family chaperone in the cytosol, and its cochaperones, DNAJA1 and DNAJA2, interact with WT α1 and α1(A322D)-containing GABA_A_ receptors (Fig. 4*A*), suggesting that Hsp70s and their Hsp40 cochaperones coordinate the folding of GABA_A_ receptors both in the ER and in the cytosol. Previously, we showed that CANX interacts with WT α1 and α1(A322D)-containing GABA_A_ receptors in a glycosylation-dependent manner (41, 52, 53). Here, we verified that UGGT1, a reglucosylation enzyme that rescues glycoproteins with minor folding defects with a client preference for large plasma membrane proteins (54, 55), interacts with α1 subunit–containing receptors (Fig. 4*A*), suggesting its potential role in folding glycosylated GABA_A_ receptors. In addition, the interaction between α1 subunit–containing GABA_A_ receptors and UNC45A, an Hsp90 cochaperone, was demonstrated (Fig. 4*A*).

Furthermore, we verified the cellular interactions between GABA_A_ receptors and three ubiquitin E3 ligases that we identified (LTN1, UBR5, and TRIM21) (Fig. 4*A*). Mammalian ubiquitin E3 ligases play a central role in the ubiquitination and targeting of their client proteins to the cellular clearance pathway (56, 57, 58). Therefore, these E3 ligases are promising candidates to direct GABA_A_ receptors to the proteasome for degradation. In addition, the interaction between α1 subunit–containing GABA_A_ receptors and ERLIN2, a potential ERAD factor, which was known to promote the degradation of inositol 1,4,5-trisphosphate receptors on the ER membrane (59), was confirmed (Fig. 4*A*).

To evaluate whether the interactions between GABA_A_ receptors and the proteostasis network components that we identified are utilized in the mammalian central nervous system, we used mouse brain homogenates to carry out coimmunoprecipitation assay. Indeed, we demonstrated the endogenous interactions between α1 subunit–containing GABA_A_ receptors and their selected interactors (Fig. 4*B*), consistent with the result that most interactors are well conserved between HEK293 cells and the nervous system (Fig. 2*C*).

We selected TRIM21 (aka RO52/SSA1), a RING-type E3 ubiquitin ligase (60), for further characterization. TRIM21 is known to play a critical role in innate immunity and cell proliferation by ubiquitinating various proteins (61, 62). TRIM21 contains an N-terminal RING-finger domain, which is responsible for its ubiquitin E3 ligase activity, a B-box domain, a coiled coil domain, and a C-terminal SPRY domain, which is responsible to bind its client proteins (63). Knocking down TRIM21 using siRNA in HEK293T cells significantly increased the total α1(A322D) protein level (Fig. 4*C*, *cf.* lane 4 to lane 3) without an apparent influence on WT α1 subunits (Fig. 4*C*, *cf.* lane 2 to lane 1), indicating that TRIM21 targets misfolded α1(A322D) subunits for degradation more favorably. Moreover, cycloheximide (CHX), a potent protein synthesis inhibitor, was applied to the cells for the indicated time to determine the degradation kinetics of α1(A322D) proteins. Compared with nontargeting siRNA control, CHX-chase experiments showed that knocking down TRIM21 increased the remaining α1(A322D) protein levels from 39% to 63% at 0.5 h post CHX application (Fig. 4*D*, *cf.* lane 2 to lane 5), and from 12% to 30% at 1 h post CHX application (Fig. 4*D*, *cf.* lane 3 to lane 6), indicating that depleting TRIM21 attenuated the degradation of α1(A322D) subunits substantially. These results supported that TRIM21 positively regulates the ERAD of the misfolding-prone α1(A322D) subunits.

## Discussion

**Figure 5:**
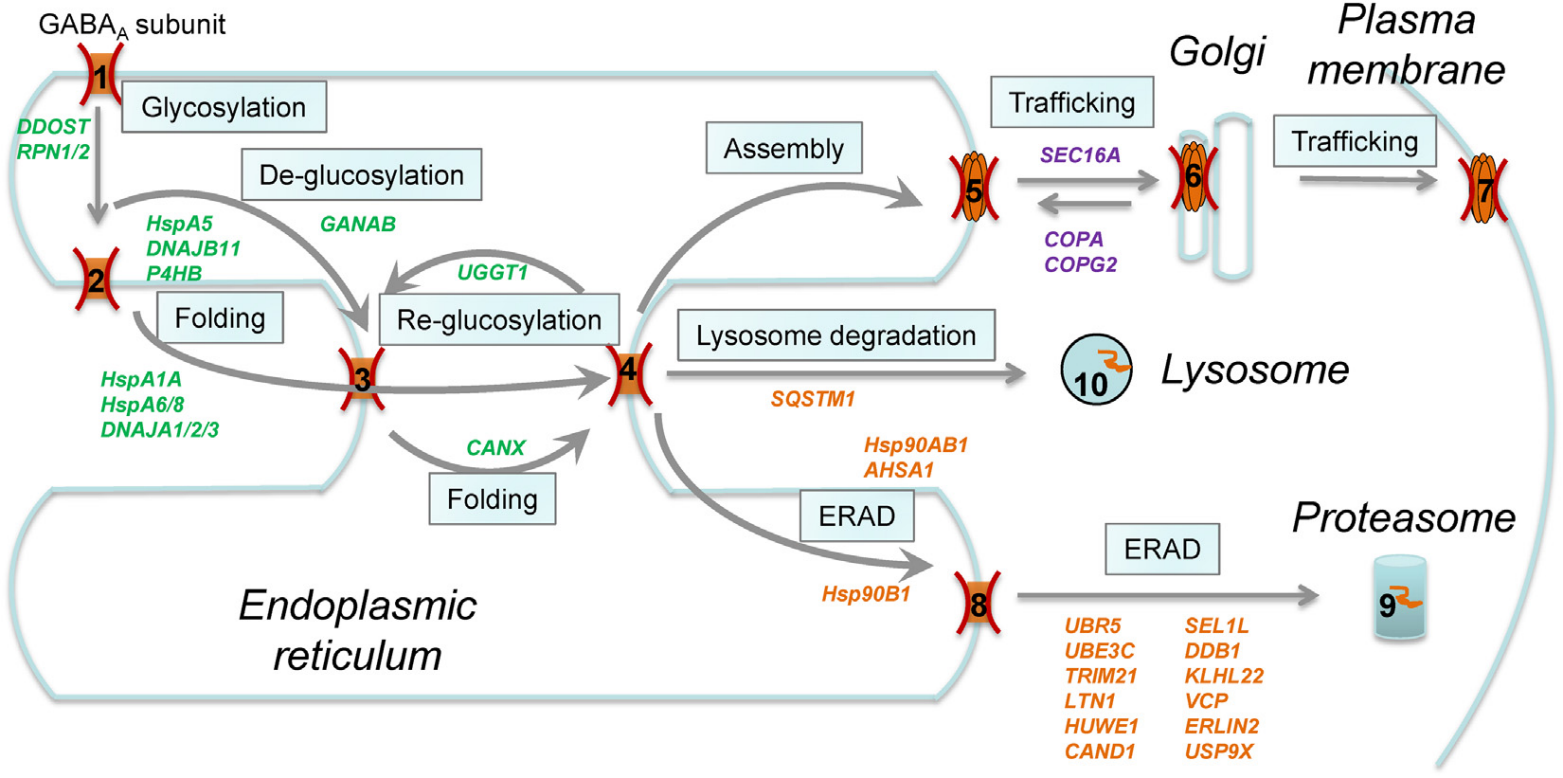
**Cellular model of the proteostasis network for GABA**_**A**_**receptors.** Interactors that were identified from our SILAC analysis are included in each step. Interactors that are expected to play a role in the folding and assembly of GABA_A_ receptors are colored in *green*; those in their degradation, in *orange*; and those in their transport, in *purple*. The GABA_A_ receptor subunit is cotranslationally translocated to the ER membrane (state 1). The OST complex, including DDOST, RPN1, and RPN2, installs *N*-linked glycans to the subunit (state 1 to state 2). The glycoprotein is trimmed by ER α-glucosidases, such as GANAB (state 2 to state 3), and subjected to calnexin (CANX) folding cycles in a glycan-dependent manner (state 3 to state 4). In parallel, heat shock proteins and their cochaperones facilitate the folding of the subunit both in the ER lumen (HspA5, DNAJB11, and P4HB) and in the cytosol (HspA1A, HspA6, HspA8, DNAJA1, DNAJA2, and DNAJA3) (state 2 to state 4). After coordinated folding efforts (state 4), the subunit faces three possible routes. *First*, if folded into the native state, the subunit assembles with other subunits to form a heteropentamer with the assistance from assembly factors (state 4 to state 5). The assembled receptors engage the COPII machinery, including SEC16A, for anterograde trafficking to the Golgi and to the plasma membrane (state 5 to state 6 and state 7). The COPI machinery, including COPA, COPG2, aids the recycle of the subunits from the Golgi back to the ER (state 6 to state 5). *Second*, non-native subunits are reglucosylated by UGGT1 for additional folding attempts (state 4 to state 3). *Third*, terminally misfolded subunits undergo ERAD pathway, being recognized, ubiquitinated, dislocated, and targeted to the proteasome for degradation (state 4 to state 8 and state 9). Ubiquitin E3 ligases (UBR5, UBE3C, TRIM21, LTN1, and HUWE1) and other ERAD factors (Hsp90B1, Hsp90AB1, AHSA1, SEL1L, VCP, and ERLIN2) potentially play a critical role in this process. Aggregated subunits could utilize the lysosome-related degradation pathway (state 4 to state 10). ER, endoplasmic reticulum; ERAD, ER-associated degradation; GABA_A_, gamma-aminobutyric acid type A; OST, oligosaccharyltransferase; SILAC, stable isotope labeling by amino acids in cell culture; UGGT1, UDP-glucose glycoprotein glucosyltransferase 1.

The comprehensive profiling of the interactomes for GABA_A_ receptors enables us to assemble a cellular model about how the proteostasis network regulates their biogenesis (Fig. 5). GABA_A_ receptor subunits are cotranslationally targeted to the ER membrane (Fig. 5, state 1), which is mediated by the SRP complex (64). The insertion of the TM domains of GABA_A_ receptors into the lipid bilayer could be facilitated by the SEC61 translocon as well as the ER membrane complex (65, 66, 67). Our SILAC analysis identified SRP68 and SSR1, which is the α subunit of a translocon-associated protein. GABA_A_ receptor subunits have several Asn *N*-linked glycosylation sites in the ER lumen, which are in the Asn-X-Ser/Thr sequence motif (X can be any residue except Pro): the α1 subunit has two sites at N38 and N138; the β2 subunit has three sites at N32, N104, and N173; and the γ2 subunit has three sites at N52, N129, and N247. The oligosaccharyltransferase complex is responsible for the transfer of 14-monosaccharide residues Glc3Man9GlcNAc2 (Glc: glucose, Man: mannose, GlcNAc: N-acetylglucosamine) to the aforementioned Asn residues in GABA_A_ receptors (Fig. 5, state 2) (68). Our SILAC analysis identified three oligosaccharyltransferase subunits, including DDOST, RPN1, and RPN2. *N*-linked glycans serve as protein maturation and QC tag (69). The *N*-linked glycan is then trimmed by α-glucosidase I and α-glucosidase II sequentially to create monoglucosylated glycan (Fig. 5, state 3). Our SILAC analysis identified GANAB, the α subunit of ER α-glucosidase II. The monoglucosylated GABA_A_ receptor subunit is the client of the membrane-bound lectin chaperone CANX for the folding process (Fig. 5, state 3 to state 4). In addition, heat shock proteins facilitate the folding of GABA_A_ receptor subunits in a glycan-independent manner both in the ER lumen and in the cytosol (Fig. 5, state 2 to state 4). Our SILAC analysis identified major Hsp60, Hsp70, and Hsp90 family chaperones as well as their cochaperones, such as HspA5 (BiP) and its cochaperone DNAJB11 (ERdj3), and HspA8 (Hsc70) and its cochaperones DNAJA1/2/3. Since each GABA_A_ subunit has a signature Cys loop in the ER lumen, protein disulfide isomerases play a critical role in assisting the formation of proper disulfide bonds. Our SILAC analysis identified P4HB, an ER protein disulfide isomerase.

After the collaborative folding efforts (Fig. 5, state 4), GABA_A_ receptor subunits have three possible pathways. *First*, if they achieve the natively folded state, they can assemble with other subunits to form a pentameric receptor for further anterograde trafficking (Fig. 5, state 4 to state 5). Properly assembled pentameric GABA_A_ receptors engage the trafficking factors to exit the ER, traffic through the Golgi, and travel en route to the plasma membrane (Fig. 5, state 5–6 and 7). Our SILAC analysis identified SEC16A in the COPII machinery that regulates the anterograde cargo protein vesicle transport from the ER to the Golgi (70) as well as COPA and COPG2 in the COPI machinery that regulates the retrograde retrieval of cargo proteins from the Golgi to the ER for recycling (71). *Second*, non-natively folded subunits are recognized by UGGT1, which was identified from our SILAC analysis. UGGT1 acts as a folding sensor and reglucosylates its substrates for re-entering the CANX folding cycles for additional folding attempts (Fig. 5, state 4 to state 3) (72). *Third*, terminally misfolded subunits are subjected to either the ERAD clearance pathway or the lysosome-related degradation pathway (73). During the ERAD, misfolded subunits are recognized, ubiquitinated, dislocated from the ER into the cytosol, and targeted to the proteasome for degradation (Fig. 5, state 4–8 and 9). Our SILAC analysis identified ubiquitin E3 ligases (HUWE1, UBR5, UBE3C, TRIM21, and LTN1) and other important ERAD factors, such as SEL1L and VCP. If misfolded subunits tend to form large aggregates in the ER, they are likely to be targeted to the lysosome for degradation (Fig. 5, state 4 to state 10). Several lysosomal degradation pathways have been described, including macroautophagy, selective ER-phagy, and direct ER-to-lysosome-associated degradation (74). Our SILAC analysis identified SQSTM1 (p62), a marker protein during autophagy by acting as an autophagosome cargo protein.

Previously, we reported that a number of proteostasis network components regulate the folding, degradation, and trafficking of GABA_A_ receptors (25, 41, 52, 75, 76), and most of them, such as BiP (HspA5), CANX, Grp94 (Hsp90B1), VCP, and SEL1L, were identified from this quantitative SILAC-based IP–MS/MS analysis, indicating the effectiveness of this proteomics approach. For example, we showed that BiP and CANX interacted with both WT α1 and α1(A322D)-containing GABA_A_ receptors; furthermore, overexpression of BiP or CANX was sufficient to enhance the ER-to-Golgi trafficking efficiency of WT α1 and α1(A322D) subunits (41). Also the interaction between CANX and α1 subunits is dependent on the *N*-linked glycosylation since mutating the glycosylation sites in α1 subunits substantially decreases such interaction (41, 53). These results indicated that BiP and CANX act as profolding chaperones to facilitate the productive folding of GABA_A_ receptors. Regarding the ERAD factors, previously we showed that Grp94 interacted with α1 subunits and that depleting Grp94 decreased ubiquitinated α1 subunits and their degradation rate (25). In addition, we demonstrated that knocking down SEL1L (a critical cofactor of the ubiquitin E3 ligase Hrd1 complex) or VCP (an ATPase that extracts misfolded proteins from the ER membrane to the cytosol) increased the total protein levels of α1(A322D) subunits (52, 76). These results supported their critical role in targeting misfolded GABA_A_ receptors to the ERAD pathway. Such knowledge assisted the integration of the interactors into the proteostasis network for GABA_A_ receptors (Fig. 5).

Here, we identified 176 interactors for α1 subunit–containing GABA_A_ receptors in HEK293T cells, including 125 interactors for WT α1-containing receptors, 105 proteins for α1(A322D)-containing receptors, and 54 overlapping interactors. In addition to three GABA_A_ subunits *(GABRA1*, *GABRB2*, and *GABRG2*), our α1 subunit–containing GABA_A_ receptor interactomes showed 13 overlapping interactors with one other GABA_A_ receptor proteomic study, including factors that regulate protein folding and degradation (*DNAJA1*, *RPN2*, *SQSTM1*, *USP9X*, *DDB1*, and *PSMC2*), transporters (*SLC25A3*, *SLC25A4*, and *SLC25A5*), an enzyme (*PTPLAD1*), and miscellaneous proteins (*PHB2*, *IMMT*, and *EMD*) (32, 36). Among the 160 interactors that were not reported from previous proteomic analyses, previously two chaperones (*HspA5* and *CANX*) were demonstrated to facilitate the folding of GABA_A_ receptors (41, 53), and three ERAD factors (*Hsp90B1*, *VCP*, and *SEL1L*) were shown to promote the ERAD of GABA_A_ receptors (25). Therefore, 155 novel interactors were identified from this study for α1-containing GABA_A_ receptors. Literature mining enabled us to assemble a cellular model about how the proteostasis network could regulate the folding, degradation, and trafficking of GABA_A_ receptors (Fig. 5), paving the foundation for future efforts to elucidate the biogenesis of GABA_A_ receptors in mechanistic details, which is not well understood.

Furthermore, our proteomic study identified 78 interactors with previously unknown function in protein folding, proteolysis, protein transport, and protein synthesis (Fig. 2*G* and Table S4). It would be of great interest to determine whether and how they could regulate GABA_A_ receptor proteostasis in the future. For instance, RCN2, a Ca^2+^-binding protein in the ER lumen, is abundant in the central nervous system, and its gene expression is upregulated in patients with idiopathic absence epilepsies (49). Dedicator of cytokinesis protein 7 (DOCK7), which is localized to the developing axons, activates Rac1 and Rac3 small GTPases and regulates neuronal polarity; moreover, its variants cause developmental and epileptic encephalopathy 23 (DEE23) (77, 78). SLC39A7 (ZIP-7) is a Zn^2+^ efflux transporter in the ER membrane; ZIP7 depletion causes neurodevelopmental impairments, and ZIP7 signaling pathway provides neuroprotective effects in recurrent seizures (79, 80).

An apparent limitation of our current proteomic study is the expression of exogenous GABA_A_ receptors in HEK293T cells, which is not within their native neuronal environment in the central nervous system. As such, some synaptic proteins were not recognized as interactors for GABA_A_ receptors. Nonetheless, since the proteostasis network is well conserved between species and tissues (81) and the majority of GABA_A_ receptor interactors have comparable protein abundance levels between HEK293T cells and human brain (Fig. 2*C*), our proteomic study should be able to identify the main components of GABA_A_ receptor interactomes. Indeed, endogenous interactions were demonstrated for selected interactors and GABA_A_ receptors in mouse brain homogenates (Fig. 4*B*). For future efforts, it would be desirable to mimic the native neuronal environment of GABA_A_ receptors by generating human-induced pluripotent stem cells carrying WT or pathogenic variant–containing receptors and differentiating such induced pluripotent stem cells into GABAergic neurons (82).

Loss of function of GABA_A_ receptors is a prominent cause of genetic epilepsies, and recent advances in genetics have identified an increasing number of GABA_A_ variants that are associated with epilepsy (16, 19). Current treatments for epilepsy focus on relieving symptoms instead of targeting the underlying causes, leaving behind much unmet medical needs. Recently, we demonstrated that adapting the ER proteostasis network is a promising strategy to restore the functional surface expression of pathogenic GABA_A_ variants (52, 83). Therefore, identification of the proteostasis network for GABA_A_ receptors paves the foundation to adjust the cellular folding, assembly, degradation, and trafficking pathways to fine-tune the functional surface expression of GABA_A_ receptors as a novel therapeutic strategy to ameliorate epilepsy. Furthermore, since WT receptors and the clinical variant-containing GABA_A_ receptors have substantially differentiating cellular interacting networks, it is feasible to adapt the proteostasis network to selectively target pathogenic variants to restore their function with minimal disruption of WT proteins. For example, here we demonstrated that depleting TRIM21, an E3 ubiquitin ligase, attenuated the degradation of α1(A322D) variant without an apparent effect on WT receptors, indicating that inhibiting ERAD factors that interact preferentially with pathogenic variants can specifically prevent the excessive degradation of variant-containing receptors. Moreover, previously we showed that small-molecule proteostasis regulators, such as stress-independent activators of the ATF6 arm of the unfolded protein response, selectively rescued the functional surface expression of misfolding-prone GABA_A_ variants by enhancing cellular folding capacity (84). Therefore, adapting the proteostasis network to specifically correct the function of pathogenic GABA_A_ receptors is a promising strategy to be further developed for the potential treatment of genetic epilepsy.

## Experimental procedures

### Plasmids

The pCMV6 plasmids containing human GABA_A_ receptor α1 subunit (UniProt no.: P14867-1) (catalog no.: RC205390), β2 subunit (isoform 2, UniProt no.: P47870-1) (catalog no.: RC216424), γ2 subunit (isoform 2, UniProt no.: P18507-2) (catalog no.: RC209260), and pCMV6 Entry Vector plasmid (pCMV6-EV) (catalog no.: PS100001) were purchased from Origene. The missense mutation A322D in the GABA_A_ receptor α1 subunit was constructed using a QuikChange II site-directed mutagenesis Kit (Agilent Genomics; catalog no.: 200523), and the complementary DNA sequences were confirmed by DNA sequencing.

### Antibodies

The mouse monoclonal anti-GABRA1 antibody (catalog no.: MAB339) was obtained from Millipore. The rabbit polyclonal anti-DNAJB11 (catalog no.: GTX105619) antibody was obtained from GeneTex. The rabbit polyclonal anti-ERLIN2 antibody (catalog no.: PA5-21736) was obtained from Thermo Fisher Scientific. The rabbit polyclonal anti-LTN1 antibody (catalog no.: AP53685PU-N) was obtained from Novus Biologicals. The rabbit polyclonal anti-DNAJA1 antibody (catalog no.: AP5849C) and anti-HspA8 (catalog no.: AP2872A) antibody were obtained from Abgent. The rabbit polyclonal anti-DNAJA2 antibody (catalog no.: 12236-1-AP), anti-TRIM21 antibody (catalog no.: 12108-1-AP), and anti-UNC45A (catalog no.: 15479-1-AP) antibody were obtained from Proteintech. The rabbit monoclonal anti-UGGT1 antibody (catalog no.: 3543-1) was obtained from Epitomics. The mouse monoclonal anti-β-actin antibody (catalog no.: A1978) was obtained from Sigma. The rabbit polyclonal anti-UBR5 antibody (catalog no.: A300-573A) was obtained from Bethyl Laboratories. The light chain–specific goat antimouse secondary antibody (catalog no.: 115-035-174) was purchased from Jackson ImmunoResearch.

### Cell culture and transfection

HEK293T cells (American Type Culture Collection; catalog no.: CRL-3216) were maintained in Dulbecco’s modified Eagle's medium (DMEM) (Fisher; catalog no.: SH3024301) with 10% heat-inactivated fetal bovine serum (FBS; Fisher; catalog no.: SH3039603HI), and 1% penicillin–streptomycin (Fisher; catalog no.: SV30010) at 37 °C in 5% CO_2_. Monolayers were passaged upon reaching confluency with 0.05% trypsin protease (Fisher; catalog no.: SH30236.01). HEK293T cells were grown in 6-well plates or 10-cm dishes and allowed to reach ∼70% confluency before transient transfection using TransIT-2020 (Mirus; catalog no.: MIR 5400) or siRNA treatment (50 nM) using the HiPerfect Transfection Reagent (Qiagen; catalog no.: 301707), according to the manufacturer’s instruction. The siRNA duplexes were obtained from Dharmacon: TRIM21 (catalog no.: J-006563-10-0005) and nontargeting siRNA (catalog no.: D-001810-01-20) as negative control. Forty-eight hours post-transfection, cells were harvested for further analysis.

HEK293T cells stably expressing either WT α1β2γ2 or α1(A322D)β2γ2 GABA_A_ receptors were generated using the G-418 selection method. Briefly, cells were transfected with α1:β2:γ2 (0.3 μg:0.3 μg:0.3 μg) or α1(A322D):β2:γ2 (0.3 μg:0.3 μg:0.3 μg) plasmids in 6-well plates. Forty-eight hours post-transfection, cells were selected in DMEM with 10% FBS and 1% penicillin–streptomycin supplemented with 0.8 mg/ml G418 for 15 days. Afterward, cells were maintained in DMEM with 10% FBS and 1% penicillin–streptomycin supplemented with 0.4 mg/ml G418. The G418-resistant polyclonal cells stably expressing GABA_A_ receptors were used for experiments.

### SDS-PAGE and Western blot

Cells were harvested with 0.05% trypsin protease (Fisher; catalog no.: SH30236.01) and then lysed with the lysis buffer (50 mM Tris, pH 7.5, 150 mM NaCl, and 1% Triton X-100) supplemented with cOmplete Protease Inhibitor Cocktail (Roche; catalog no.: 4693159001). Cell lysates were cleared by centrifugation (15,000*g*, 10 min, 4 °C), and the supernatants were collected as total proteins. Protein concentration was determined by MicroBCA assay (Pierce; catalog no.: 23235). Equal amounts of total proteins were separated in 8% reducing SDS-PAGE gels, and Western blot analysis was performed using appropriate antibodies.

### Mouse brain homogenization

C57BL/6J mice (Jackson Laboratory) at 6 to 10 weeks were sacrificed, and the cortex was isolated and homogenized in the homogenization buffer (25 mM Tris, pH 7.6, 150 mM NaCl, 1 mM EDTA, and 2% Triton X-100) supplemented with the Roche cOmplete Protease Inhibitor Cocktail. The sample was centrifuged at 800*g* for 10 min at 4 °C. The pellet was rehomogenized in the same homogenization buffer and centrifuged at 800*g* for 10 min at 4 °C. The combined supernatants were placed on a rotating device for 2 h at 4 °C and then centrifuged at 15,000*g* for 30 min at 4 °C. The resulting supernatant was collected as mouse brain homogenate, and its protein concentration was determined by a MicroBCA assay. These animal studies were approved by the Institutional Animal Care and Use Committee at Case Western Reserve University and carried out in agreement with the recommendation of the American Veterinary Medical Association Panel on Euthanasia.

### Immunoprecipitation

For immunoprecipitation using cell lysates (500 μg) and the mouse brain homogenates (1 mg), they were precleared with 30 μl of protein A/G plus-agarose beads (Santa Cruz; catalog no.: sc-2003) and 1.0 μg of normal mouse immunoglobulin G (Santa Cruz; catalog no.: sc-2025) for 1 h at 4 °C to remove nonspecific binding proteins. The precleared cell lysates were incubated with 2.0 μg of mouse anti-α1 antibody for 1 h at 4 °C and then with 30 μl of protein A/G plus agarose beads overnight at 4 °C. Afterward, the beads were collected by centrifugation at 8000*g* for 30 s and washed three times with lysis buffer. The complex was eluted by incubation with 40 μl of 2× Laemmli sample buffer (Bio-Rad; catalog no.: 1610737) in the presence of DTT. The immunopurified eluents were separated in 8% SDS-PAGE gel, and Western blot analysis was performed using appropriate antibodies.

### SILAC-based quantitative proteomics analysis

#### SILAC labeling

SILAC is an *in vivo* labeling strategy for MS-based quantitative proteomics (38, 85). HEK293T cells stably expressing either WT α1β2γ2 or α1(A322D)β2γ2 GABA_A_ receptors were labeled with heavy media (SILAC DMEM media [Pierce; catalog no.: 88420] plus 10% dialyzed FBS [Sigma; catalog no.: F0392], 1% penicillin–streptomycin, 0.01% ^13^C_6_ l-lysine–2 HCl [Pierce; catalog no.: 89988], 0.01% ^13^C_6_ l-arginine–HCl [Pierce; catalog no.: 88210], and 0.002% l-proline [Pierce; catalog no.: 88430]), whereas HEK293T cells that were transfected with EV plasmids were cultured in normal light media (SILAC DMEM media plus 10% dialyzed FBS, 1% penicillin–streptomycin, 0.01% l-lysine–2 HCl (Pierce; catalog no.: 88429), 0.01% l-arginine–HCl (Pierce; catalog no.: 88427), and 0.002% l-proline) for 14 days to ensure complete labeling.

#### Coimmunoprecipitation and in-gel digestion

Cells were then harvested with 0.05% trypsin and lysed in the lysis buffer (50 mM Tris, pH 7.5, 150 mM NaCl, and 1% Triton X-100) supplemented with Roche cOmplete Protease Inhibitor Cocktail. Lysates were cleared by centrifugation (15,000*g*, 10 min, 4 °C). Protein concentration was determined by MicroBCA assay. The same amount of light and heavy cell lysates was mixed by 1:1. Six milligrams of total proteins were immunoprecipitated using a mouse monoclonal anti-GABA_A_ receptor α1 subunit antibody. The immunoisolated complexes were separated by reducing SDS-PAGE and stained with Coomassie blue to visualize protein gel bands (Fig. 1*C*). The gel was washed in distilled water to remove excess background stain. The gel was then divided to six parts evenly, excised, and destained with 500 μl of 1:1 acetonitrile (ACN) and 100 mM ammonium bicarbonate (ABC) solution for 2 to 8 h. Afterward, 10 mM reductive Tris(2-carboxyethyl)phosphine was added for 30 min, and then free cysteines were alkylated with 55 mM iodoacetamide for 20 min in the dark. ACN and 100 mM ABC were used to dehydrate and rehydrate the gel pieces alternatively for three times. Gel pieces were swelled in 50 mM ABC containing freshly prepared 10 ng/μl trypsin (Promega, sequencing grade) and digested overnight. Peptides were extracted with 60% ACN/5% formic acid (FA). Digested peptides were cleaned through C18 spin columns (Thermo Pierce), dried in SpeedVac, and saved at −80 °C if not immediately analyzed.

#### Tandem MS analysis

Three biological replicates were analyzed by LC–MS/MS. Digested peptides were reconstituted with 0.1% FA and analyzed by LC–MS/MS. Separation of peptides *via* capillary liquid chromatography was performed using Waters nanoAquity system (Waters Corp). Mobile phase A (aqueous) contained 0.1% FA in 5% ACN, and mobile phase B (organic) contained 0.1% FA in 85% ACN. Separation was achieved using a C18 column (75 μm × 20 cm; Waters Corp; Ethylene Bridged Hybrid column; #BEH300) through a 300-min gradient of 6% to 45% mobile phase B at a flow rate of 300 nl/min. MS analysis was performed using a hybrid linear ion trap Orbitrap Velos mass spectrometer (LTQ-Orbitrap Velos; Thermo). Survey scans were operated at 60,000 resolution, followed by 20 collision-induced dissociation fragmentations.

#### Database search

Acquired tandem mass spectra were searched against UniProt human protein database with 20,244 protein entries, downloaded on May 5, 2011. A decoy database containing the reversed sequences of all proteins was appended to estimate the FDR (86). Protein identification using SEQUEST (87) or ProLuCID (88) and DTASelect (89, 90) and quantification using Census (91) were performed using the Integrated Proteomics Pipeline (IP2; Integrated Proteomics Applications). Mass accuracy was limited to 10 ppm for precursor ions and 0.6 Da for product ions, with tryptic enzyme specificity and up to two missed cleavages. Static modifications included carbamidomethylation on cysteines (57 Da), and variable modifications included oxidation on methionines (16 Da) in addition to the SILAC modifications, that is, heavy ^13^C_6_ on lysines and arginines (6 Da). DTASelect (89, 90) was applied to generate search results for peptide-to-spectra matches with a maximum FDR of 5%, yielding a protein FDR of less than 1%. Protein quantification based on SILAC peptide pairs was performed with Census (91). Census allows users to filter peptide ratio measurements based on a correlation threshold because the correlation coefficient (values between zero and one) represents the quality of the correlation between the unlabeled and labeled chromatograms and can be used to filter out poor quality measurements. In this study, only peptide ratios with correlation values greater than 0.5 were retained. High-confidence protein quantifications were based on two or more SILAC pairs for a given protein.

The interactomes of WT α1 and α1(A322D)-containing GABA_A_ receptors were identified using the SILAC ratio with arbitrary yet strict criteria to remove potential false positives. Only proteins with *p* < 0.05 were further considered. To be included as an interactor, it must (1) have a SILAC ratio of WT α1/EV or α1(A322D)/EV to be at least 1.30 and (2) have a Benjamini and Hochberg correction (42) of FDR of no more than 0.10. For display purpose in Figure 3, *B* and *C*, if the SILAC ratio is greater than 4.0, it is displayed as 4.0; if a protein is only detected in one sample, the SILAC ratio of this protein in the other sample is artificially set to 1.0. The final list of the interactomes of α1 subunit–containing GABA_A_ receptors and α1(A322D)-containing GABA_A_ receptors is presented in Table S1.

### GO analysis

Cellular component and biological process of GABA_A_ receptor interactomes were analyzed using DAVID (https://david.ncifcrf.gov/) (46, 47).

Protein abundance analysis was performed using the ProteomicsDB database (https://www.proteomicsdb.org/) (43). RNA abundance analysis was carried out using the Human Protein Atlas database (https://www.proteinatlas.org/) (44, 45).

## CHX-chase assay

HEK293T cells stably expressing α1(A322D)β2γ2 GABA_A_ receptors were seeded at 2.5 × 10^5^ cells per well in 6-well plates and incubated at 37 °C overnight. Cells were then transfected with indicated siRNA. Forty-eight hours post transfection, cells were incubated with CHX (100 μg/ml) (Enzo; catalog no.: ALX-380-269-G001), a potent protein synthesis inhibitor, to stop protein translation, and chased for the indicated time. Cells were then harvested and lysed for SDS-PAGE and Western blot analysis.

## Statistical analysis

All data are presented as mean ± SD. Statistical significance was calculated using Student’s *t* test for two-group comparison. If more than two groups were compared, ANOVA followed by post hoc Tukey's test was used. A *p* value of less than 0.05 was considered statistically significant. ∗*p* < 0.05 and ∗∗*p* < 0.01.

## Data availability

All data are contained within the article.

## Supporting information

This article contains supporting information (six supporting tables).

## Conflict of interest

The authors declare that they have no conflicts of interest with the contents of this article.

## Supporting information

Table S1 List of proteins and their SILAC ratios from the quantitative proteomics experiments to identify GABA_A_ receptor interactomes. Labeled with a unique entry number for each protein, first 54 proteins overlap within WT α1-containing GABA_A_ receptor interactome and the α1(A322D)-containing GABA_A_ receptor interactome. Number 55 to 125 are WT α1-containing GABA_A_ receptor only interactors, whereas number 126 to 176 are α1(A322D)-containing GABA_A_ receptor only interactors

Table S2 List of protein abundance and RNA abundance of GABA_A_ receptor interactors. Scores of protein abundance were retrieved from the ProteomicsDB database (https://www.proteomicsdb.org/). Protein comparison included data from HEK293 cells and human brain tissues. Scores of RNA abundance were retrieved from the Human Protein Atlas database (https://www.proteinatlas.org/). RNA comparison included data from HEK cells, SH-SY5Y cells and human brain tissues. Fpkm, fragments per kilobase of exon per million mapped fragments

Table S3 List of cellular location and membrane proteins of GABA_A_ receptor interactors. Entry number for each protein was the same as in Table S1 for overlapping, WT α1-containing GABA_A_ receptor only, or α1(A322D)-containing GABA_A_ receptor only interactors. In the column of *Membrane protein*, *1* means that the corresponding protein is indeed an integral membrane protein, whereas *0* means otherwise. Moreover, cellular location statistics and membrane protein statistics were presented as well, summarizing number of interactors in each GO term

Table S4 List of biological process for GABA_A_ receptor interactors. Entry number for each protein was the same as in Table S1 for overlapping, WT α1-containing GABA_A_ receptor only, or α1(A322D)-containing GABA_A_ receptor only interactors. In the column of *Function 2*, further available information was provided. Moreover, biological process statistics were presented as well, summarizing number of interactors in each category

Table S5 List of enriched biological process for GABA_A_ receptor interactors. Data were analyzed and retrieved from Database for Annotation, Visualization and Integrated Discovery (DAVID). Five functional clusters were presented from both WT α1-containing GABA_A_ receptor and α1(A322D)-containing GABA_A_ receptor interactors. GO terms, enriched genes, and FDR were included

Table S6 List of the proteostasis network components among GABA_A_ receptor interactors. Based on GO analysis and literature mining, three categories were included as the following: protein folding, proteolysis, and protein transport, for overlapping, WT α1-containing GABA_A_ receptor only, or α1(A322D)-containing GABA_A_ receptor only interactors
